# Incidental Non-cardiac Findings in Cardiovascular Imaging

**DOI:** 10.1007/s11936-018-0700-5

**Published:** 2018-10-23

**Authors:** Mark T. Macmillan, Michelle C. Williams

**Affiliations:** 1 Department of Radiology, Royal Infirmary of Edinburgh, Edinburgh, UK; 2 University of Edinburgh/BHF Centre for Cardiovascular Science, Chancellor’s Building, SU305, 49 Little France Crescent, Edinburgh, EH16SUF UK; 3 Edinburgh Imaging facility QMRI, University of Edinburgh, Edinburgh, UK

**Keywords:** Imaging, Incidental non-cardiac findings, Cardiac imaging

## Abstract

Improvements in imaging techniques have led to an expansion in the number of cross-sectional cardiac studies being performed. This means that incidental non-cardiac findings (INCF) identified on cardiac imaging have become an important clinical concern. The majority of INCF are not clinically significant. However, some INCF will require follow-up or changes in management. Differentiating clinically significant from non-significant INCF can be challenging, particularly given the breadth of potential findings and the range of organ systems involved. Following up INCF also has economic implications. Recent changes to the lung nodule follow-up guidelines will reduce the cost of following up incidental lung nodules. In this manuscript, we discuss the common and important INCF which may be identified in cardiovascular imaging and explore potential implications of these findings.

## Introduction

Technological advances have allowed the development of computed tomography (CT), magnetic resonance imaging (MRI) and nuclear imaging methods to assess the heart and coronary arteries. However, the increasing use of cross sectional imaging has also led to increased detection of incidental non-cardiac findings (INCF). These findings can affect any organ in the imaged field-of-view, with lung abnormalities being the most common. The identification of INCF is important in cardiac imaging in order to identify alternative causes of symptoms or previously undiagnosed conditions that require investigation or treatment. The detection of INCF may also have important implications for patients and may necessitate additional imaging, consultations, follow-up or treatment.

### Frequency of INCF

INCF can be defined as abnormalities which are potentially clinically relevant and are identified despite being unrelated to the purpose of the investigation [1]. They can be classified as benign or clinically significant, with clinically significant findings being those which cause symptoms or require further investigation or management [2•]. In the context of cardiac CT it is often difficult to strictly differentiate between INCF which are truly incidental and those that may be the cause of the patient’s symptoms of chest pain or breathlessness. This is one reason for differences in the prevalence of INCF between studies.

INCF are frequent in all forms of cardiac imaging; in cardiac CT, approximately 44% of patients will have at least one non-cardiac finding [3••], in cardiac MRI 35% [4] and in cardiac SPECT 55% [5]. The rate of clinically significant INCF is between 10 and 17% [2•, 3••, 6–8, 9••, 10]. In a meta-analysis of 11,703 patients in 13 studies, acutely life-threatening INCF were identified on CT in 2.2% and malignancy was identified in 0.3% [9••]. In a meta-analysis of 5082 patients in 10 studies, the frequency of malignancy on CT was estimated as 0.7% [3••]. Similar rates of malignancy have been identified in the low-dose CT performed for attenuation correction in SPECT imaging [11].

The type of imaging performed impacts the prevalence of INCF as they are least common in non-contrast CT for calcium scoring and more common in CT performed to assess bypass grafts or pulmonary veins [12]. In nuclear imaging, INCF may be identified where the abnormality causes an increase or decrease in the normal uptake, particularly in organs involved in tracer excretion. If cross-sectional vascular imaging is performed prior to structural interventions such as transcutaneous aortic valve implantation (TAVI), then more INCF will be identified due to the larger scan range and potentially older population. The frequency of clinically significant INCF in pre-TAVI patients is 17 to 18% [13, 14]. One study found that the identification of INCF in these patients is associated with a lower chance of TAVI being performed and a worse overall outcome [15]. However, other studies have found that after multivariate analysis, outcomes after TAVI were not influenced by INCF [14] and that 2 year outcomes were similar after a decision to perform the TAVI was made [16].

In addition to this substantial prevalence, INCF can be identified within any organ within the imaged field-of-view. Nevertheless, imaging optimised for cardiac assessment may not be optimal for assessing other organs and thus may under- or overestimate the presence of abnormalities in other organs. Therefore, a diverse range of INCF are possible, and a wide breadth of knowledge is required to read these examinations and recommend suitable subsequent investigations or follow-up for INCF.

### Lungs

**Fig. 1:**
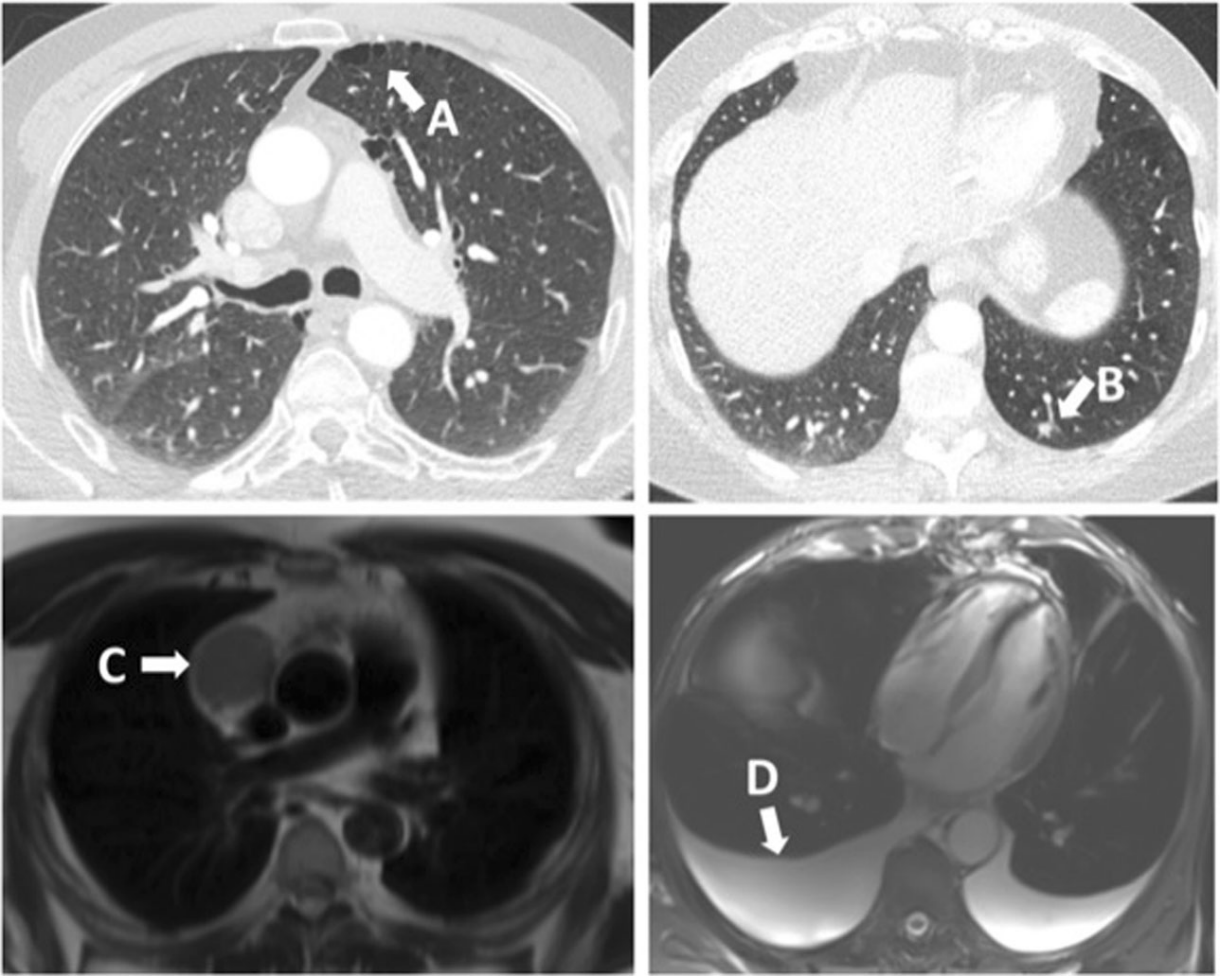
CT and MRI images of common INCF (A, paraseptal emphysema on CT; B, 7-mm pulmonary nodule on CT which requires follow-up imaging; C, mediastinal lymphadenopathy on MRI; D, pleural effusion on MRI).

The lungs are the most common site for INCF on cardiac imaging, largely due to the prevalence of pulmonary nodules and emphysema (Table 1, Fig. 1) [6, 7]. Lung nodules occur in 14% of patients undergoing coronary CT angiography [17]. Many of these require no further investigation or treatment, but some may represent important treatable malignancy or require further follow-up imaging.

**Table 1:** Common INCF identified on cardiovascular imaging

Organ	Finding	
Lung	Lung nodules	Benign nodules, intrapulmonary lymph nodes, calcified granulomata, malignant nodules or masses
	Parenchymal abnormalities	Emphysema, pulmonary fibrosis bronchiectasis
	Infections	Bacterial pneumonia, tuberculosis, atypical infections
	Pleural abnormalities	Pleural effusion, pleural plaques, pleural malignancy
Mediastinum	Lymphadenopathy	
	Mediastinal mass	Thymoma, teratoma, lymphoma, germ cell malignancy, benign cyst
	Thoracic aorta	Atherosclerosis, aneurysm, dissection
Neck	Thyroid	Cyst, nodule, malignancy
	Lymphadenopathy	
Abdomen	Liver	Cyst, haemangioma, malignancy, fatty infiltration, cirrhosis, ascites
	Gallbladder	Calculi, cholecystitis, malignancy
	Kidneys	Cyst, malignancy, calculi, scarring
	Pancreas	Calcification, cyst, malignancy, atrophy
	Spleen	Enlargement, cyst, malignancy
	Stomach	Malignancy
	Large and small bowel	Malignancy, diverticulosis, hernia
	Adrenals	Benign adenoma, metastasis, primary malignancy
	Pelvic organs	Uterus, ovary, prostate, testes, bladder
Bones	Benign lesion	Haemangioma, bone island
	Malignant lesion	Metastasis, primary malignancy
	Degenerative lesion	Osteoarthritis, compression fracture

In cardiac CT, the lungs should be assessed on both scout images and wide field-of-view images reconstructed with a dedicated lung reconstruction algorithm. Cardiac CT images usually cover only the lower chest in order to minimise radiation dose, so abnormalities in the upper zones may only be visible on scout images. More INCF will be identified on wide field-of-view imaging, as 54% of the total lung volume is visualised compared to 14% on cardiac field-of-view images [3••, 18]. Indeed, 80% of pulmonary nodules seen on the wide field-of-view images are not seen on the limited cardiac field-of-view [19].

Similar abnormalities may be identified on the CT performed for attenuation during SPECT or PET imaging. Some malignancies may also take up radiotracers during SPECT imaging, such as carcinoid tumours [20, 21]. During cardiac MRI, lung abnormalities may be identified on any sequences, but in particular, axial and coronal images should be reviewed for the presence of INCF. One study found that INCF were easier to identify on balanced steady state free precession (bSSF) images but that T1w-half-fourier acquisition single-shot turbo spin-echo (HASTE) could provide additional diagnostic information [22].

Pulmonary nodules represent a challenge in cardiac imaging as, although many are benign, it is important to identify nodules with a risk of malignancy and organise suitable follow-up. Guidelines for the follow-up of lung nodules have recently been updated with the 2017 Fleischner society guidelines [23] and the 2015 British Thoracic Society guidelines [24]. The 2005 Fleischner guidelines recommended routine follow-up imaging for nodules greater than 4-mm diameter with the interval determined by nodule size, characteristics and clinical risk factors [25]. This lead to a significant burden of follow-up imaging after cardiac CT [2•]. The revised 2017 Fleischner guidelines increase the threshold for follow-up imaging to 6 mm or 100 mm^3^ [23]. Part-solid or “ground glass” nodules require follow-up for longer due to the risk of in situ and low-grade adenocarcinoma [26].

Whilst the majority of pulmonary nodules are found to be clinically insignificant, there is a subgroup which represent malignant disease. The prevalence of malignancy in patients undergoing cardiac CT is estimated at 0.7%, and 72% of these are attributed to lung cancer [3••]. In comparison, the baseline prevalence of lung cancer was 1% in the National Lung Screening Trial (NLST) [27] and 1.7% in the UK Lung Cancer Screening (ULCS) pilot trial [28]. The NLST compared the use of low-dose CT and chest radiographs and demonstrated a 20% relative reduction in mortality in the CT group [29]. The ULCS pilot trial demonstrated that it was possible to detect lung cancer earlier and provide curative therapy in 80% of cases [28]. Considering the small difference in prevalence between cardiac CT studies and those selected for lung cancer screening, it is not unreasonable to postulate that detection and follow-up of pulmonary nodules detected by cardiac CT may similarly reduce mortality.

INCF which require acute intervention may also be identified in the lungs, particularly on contrast-enhanced cardiac CT. The pulmonary arteries are often opacified to some degree during cardiac CT, which allows for detection of pulmonary embolism. Analysis of incidental findings in the SCOT-HEART study identified pulmonary embolism in 0.2% of patients ^6^. Whilst this is a relatively low proportion, the morbidity and mortality associated with pulmonary embolism are significant. Similarly, pneumonia, including atypical infections, is a treatable condition which may be identified on cardiac imaging.

Cardiac imaging may also identify previously unknown chronic lung conditions such as emphysema or pulmonary fibrosis. There is an overlap between COPD and coronary artery disease in terms of risk factors such as smoking and pathological mechanisms such as inflammation [30]. Therefore, the frequent identification of emphysema on cardiac CT is not unexpected. Depending on the phase of respiration and use of iodinated contrast, it may not be possible to identify subtle parenchymal abnormalities, as these may be obscured on images obtained in expiration or with contrast. The identification of pulmonary fibrosis can be challenging on cardiac CT performed with contrast enhancement, and subsequent non-contrast CT imaging may be required to assess the lung parenchyma. Subtle changes of lung parenchymal abnormalities may also be identified on raw SPECT images, such as the flattening of the diaphragm in patients with COPD or reduced counts in patients with pleural effusions [20].

### Mediastinum

Enlarged mediastinal lymph nodes are a common INCF in both cardiac MRI and CT, and may be identified in 2.4 to 4.7% of patients undergoing MRI [6, 31] and 1.7% of patients undergoing cardiac CT [2•]. These lymph nodes can indicate underlying malignancy or inflammation, and therefore are essential to detect and interpret. In general, mediastinal lymph nodes which are greater than 10 mm in the shortest axial diameter are considered abnormal. However, nuances exist within different nodal stations [32] as normal subcarinal lymph nodes may measure up to 2 cm and normal right lower paratracheal nodes can measure up to 1.5 cm [33]. Conversely retro-crural and epicardial lymph nodes may be abnormal at much smaller diameters [34]. Mediastinal masses may also be identified during cardiac imaging. These may represent benign pericardial, bronchogenic or oesophageal duplication cysts. However, malignant pathologies such as teratoma, thymoma, thyroid malignancy, germ cell tumours and lymphoma can also be identified.

Depending on the portion of the chest imaged, the ascending, arch and descending thoracic aorta may be visible on cardiac imaging. A discussion of the significance of incidental aortic atherosclerosis is out with the scope of this article but may represent a clinically important finding and is associated with prognosis [35]. Incidental thoracic aortic aneurysms can be identified on CT or MRI imaging, as are vascular anomalies which could be important for subsequent invasive investigations. Intramural haematoma may be identified as high attenuation within the wall of the aorta on non-contrast CT imaging. Aortic dissection is a potentially lethal INCF which may be identified in patients with acute chest pain. In a study of over 11,000 patients undergoing cardiac CT for acute chest pain, 1.1% has an aortic dissection [36].

Hiatus hernia is a very common INCF which may be identified on cardiac CT and MRI. Although this may be an alternate cause for the patient’s symptoms, they may also be asymptomatic. In our centre, the identification of hiatus hernia on cardiac CT was not associated with the instigation of gastric acid suppression therapy [2•]. The oesophagus is difficult to assess on cardiac CT, but oesophageal masses may be identified and endoscopy would be required to assess these further.

### Neck

The lower neck is often included as part of localising images on cardiac MRI or the scout images performed during cardiac CT. The most common pathologies identified are thyroid nodules and lymph node enlargement. Lymph nodes may also be identified in the supraclavicular fossa on MRI or if particularly large on the scout images performed prior to cardiac CT. Similar to lymph nodes in the thorax, nodules less than 1 cm diameter can be dismissed in the majority of cases.

Thyroid enlargement and thyroid nodules are frequently encountered when reading cardiac MRI examinations [37]. The American Society of Thyroid Disease strongly recommends clinical history and examination, thyroid stimulating hormone (TSH) blood test and ultrasound for all cases of thyroid nodule enlargement [38]. In cases with low TSH, a nuclear imaging study of the thyroid is also recommended [38]. In theory, thyroid nodules should generate a significant burden of imaging and clinical follow-up, but in practice, this is not apparent. Grady et al. demonstrated variability in the practice of reporting thyroid nodules, even in cases of nodules greater than 20 mm in diameter [39]. A conservative approach is often appropriate when assessing thyroid nodules, and local guidelines should be followed.

### Abdomen

Sections of the liver, spleen, pancreas, adrenals, kidneys, stomach and bowel may be imaged on cardiac CT and MI. These findings make up a significant portion of INCF, and many require no further follow-up or treatment. In cardiac CT and MRI, only a small proportion of the upper abdomen is imaged. However, in CT assessment prior to TAVI, the larger scan range will include the whole of the abdomen and pelvis.

The commonest intra-abdominal INCF are hepatic cysts, accounting for 5% INCF [2•]. Simple hepatic cysts, which have uniform fluid attenuation, no visible wall and no contrast enhancement, are benign and require no further imaging. In cases where the cysts do not clearly meet these criteria, further assessment with ultrasound or MRI may be required. Other benign liver abnormalities that can be identified include haemangioma and fatty infiltration [2•]. Malignancy including metastases or primary malignancy may also be identified. Subtle abnormalities may be identified in the liver during SPECT imaging such as reduced radiotracer uptake in patients with cirrhosis, multiple liver cysts or malignancy [20].

The kidneys are often not fully imaged on cardiac imaging, but the upper poles are usually included on MRI and may be identified on cardiac CT. Renal cysts are a common finding and are frequently benign and require no further imaging. However, features such as septations, internal density, enhancement, calcification or solid components increase the chance of malignancy [40]. Renal cysts are classified according to the Bosniak criteria and should be followed up accordingly [41].

Adrenal, splenic and pancreatic lesions are less frequently identified but require diligent assessment, as all three organs are potential sites for malignant pathology. Adrenal nodules often represent benign adenoma, and an attenuation below 10 Hounsfield units on non-contrast imaging has a good diagnostic accuracy for identifying benign adenoma. Review of previous imaging may also help to identify clearly benign lesions. However, if these criteria cannot be fulfilled, further assessment with MRI or contrast enhanced CT may be required. Similarly, some pancreatic cysts can be clearly identified as benign, but further assessment or follow-up will be required if malignancy is suspected [42]. Splenic lesions are rare, but an enlarged spleen may be indicative of underlying pathologies, including haematological malignancy.

A limited proportion of the stomach and large or small bowel may be included on cardiac CT or MRI. The unprepared bowel can be difficult to assess, but these areas should be reviewed for incidental malignancy. Abnormal lymph nodes may be identified throughout the abdomen and pelvis. If the pelvis is imaged then assessment of the uterus, ovaries and adnexa is required and may identify incidental ovarian cysts or endometrial or ovarian malignancy. If these are suspected, then dedicated imaging of these regions will be required. Incidental inguinal hernia are a common INCF in the pelvis, particularly in elderly males.

### Bones and soft tissues

The most important distinction to make when assessing lesions within the bones is whether the lesion can confidently be called benign. Fortunately, some of the more common bone lesions have classic imaging characteristics which allow them to be identified. Haemangioma have a thinned trabecular pattern on CT and on MRI are most often high signal on both T1 and T2-weighted imaging. Bone islands are uniformly dense with regular margins on CT. Irregular lesion margins, wide transitional zones and bony destruction are all features that may suggest an aggressive lesion which requires further assessment.

In older patients, degenerative change and compression fractures may be identified in the thoracic spine. These may be most apparent on sagittal images. Although they may be asymptomatic, the identification of these findings may aid future management, including identifying patients who could benefit from bone protection therapy ^26^.

Metastases represent the most common malignant bone lesions, and these may be identified in the vertebrae, ribs, sternum, scapulae or clavicles on cardiac imaging. Careful review of these areas is necessary, and subsequent imaging will be required in cases with suspicious features.

Scrutiny of the skin, muscles and subcutaneous fat that has been covered by the imaging field-of-view is also required. Occasionally, metastases to these regions can aid the identification and staging of malignancy.

### Breasts

Breast cancer is one of the most common forms of cancer in females [43], and it is inevitable therefore that breast cancers will be identified incidentally on cardiac imaging. Features such as spiculation, distortion of the adjacent breast tissue and an irregular contour can help to identify malignant lesions. However, the predictive value of non-dedicated CT to identify breast malignancy is low [44]. Therefore, if suspicious lesions are identified, further review by the breast team with mammography or ultrasound should be considered. In addition to malignant INCF in the breast, several benign breast lesions are common including fibroadenoma, fat necrosis and breast cysts.

### Economic implications and psychological impact

The burden of further investigations, management and follow-up contributes to the cost and psychological impact of INCF.

The cost of INCF depends on the prevalence of abnormal findings, follow-up imaging practices and local costs. A Danish study in 2011 of 1383 patients undergoing coronary CT incurred a cost of 58 euros per patient for the assessment of INCF [45]. A smaller study in 2008 in the USA of 151 patients found a cost of $17.42 per patient for follow-up imaging, not including clinic follow-up [46]. A Canadian study which also considered costs from investigations such as lung biopsies and the associated complications found a much higher cost per patient of $59.62 per patient [47]. An Australia study identified a cost per patient of $63.62 to perform additional imaging for lung nodules [17]. In patients undergoing investigation prior to TAVI, the cost to follow-up INCF was low at £32.69 per TAVI patient (ED).

When contextualised per individual, the cost does not seem significant, but it is important to consider these costs with reference to clinical benefits and the number of cardiac imaging investigations which are now being performed. A Danish study found that the cost of INCF equated to 40,190 euros to save one life from malignancy [45]. Goehler et al. estimated that the cost per quality adjust life year for lung nodule follow-up was $154,000, which is greater than the normal accepted level from an intervention [48]. However, when considering these results, it is important to note that they were carried out prior to the updated Fleischner guidelines which reduce the number of nodules which require follow-up (Table 2). Indeed, application of these new guidelines would reduce the cost of lung nodule follow-up by 57%, from £7.04 to £3 per patient, by reducing the number of patients requiring follow-up. Further studies have demonstrated a 56% reduction in follow-up of pulmonary nodules when using the new Fleischner guidelines [49•].

**Table 2:** Fleischner society guidelines for the follow-up of lung nodules (*m* months)

Type	Number	Size	Follow-up CT	
			Low-risk patient	High-risk patient
Solid	Single	< 6 mm, < 100 mm^3^	None	(Optional 12 m)
		6–8 mm, 100–250 mm^3^	6–12 m (consider 18–24 m)	6–12 m then 18–24 m
		> 8 mm, > 250 mm^3^	CT at 3 months or PET/CT or biopsy	
	Multiple	< 6 mm, < 100 mm^3^	None	(Optional 12 m)
		6–8 mm, 100–250 mm^3^	3–6 m (consider 18–24 m)	3–6 m then 18–24 m
		> 8 mm, > 250 mm^3^	3–6 m (consider 18–24 m)	3–6 m then 18–24 m
Sub-solid	Groundglass	< 6 mm	None	
		> 6 mm	6–12 m then 3–5 years	
	Part-solid	< 6 mm	None	
		> 6 mm	3–6 m then annually for 5 years	
	Multiple	< 6 mm	3–6 m then at 2 years and 4 years	
		> 6 mm	3–6 m then based on most suspicious nodule	

Whether or not INCF lead to further follow-up is impacted by a variety of parameters related to both physicians and patients [50]. In the past, it was suggested that INCF could be ignored and only the cardiac findings reported [51]. However, current opinion is that that all imaged organs should be assessed, as to ignore INCF would deny the patient the ability to share decision making and access potential treatment. The identification of INCF may benefit patients by identifying previously un-diagnosed conditions which can be treated. However, they may also cause anxiety and the burden of further follow-up investigations, clinic consultations and treatments [52]. Nevertheless, studies have shown that patients value the opportunity to know about incidental imaging findings [53]. Good communication is important in minimising the potential distress caused by the identification of INCF [54].

## Conclusion

INCF are common in cardiac imaging and can occur within multiple anatomical areas. The wide range of INCF which can be identified illustrates the depth of knowledge and experience required to identify and interpret these findings. While many INCF are benign, an important proportion require further investigation, follow-up or treatment. Questions have been raised regarding the cost effectiveness and clinical implications of investigating INCF; however, new guidance for the management of pulmonary nodules will reduce the requirement for follow-up imaging and improve cost effectiveness. It is important that all images are reviewed for potentially clinically significant INCF, as important treatable diseases, including malignancy, may be identified.
